# Vaccination and Antiviral Combination Improves Resolution Time of COVID‐19 Infection in Treated Multiple Myeloma: A Retrospective Study

**DOI:** 10.1155/ah/5597825

**Published:** 2026-06-15

**Authors:** M. Di Cecca, Bongarzoni V., Garzia M., Fazio F., Tomarchio V., Barilà G., Liberatore C., Rago A., Rossini B., Rocco S., Pisani F., De Padua L., Fiori L., Tafuri M. T., Anaclerico B., Cardarelli L., Scomazzon E., Fioritoni F., Caravita Di Torritto T., Quinto A. M., Palmieri S., Lamanda M., Tamburini A., Piciocchi A., Petrucci M. T., Rigacci L., Annibali O.

**Affiliations:** ^1^ Unit of Hematology, Stem Cell Transplantation, Transfusion Medicine and Cellular Therapy, Department of Medicine, Università Campus Bio-Medico di Roma, Rome, Italy, unicampus.it; ^2^ Department of Hematology, San Giovanni-Addolorata Hospital, Rome, Italy, hsangiovanni.roma.it; ^3^ Hematology and Stem Cell Transplant Unit, San Camillo Forlanini Hospital, Rome, Italy, scamilloforlanini.rm.it; ^4^ Department of Translational and Precision Medicine, Hematology Azienda Policlinico Umberto I Sapienza University of Rome, Rome, Italy; ^5^ Unit of Hematology, Stem Cell Transplantation, Transfusion Medicine and Cellular Therapy, Fondazione Policlinico Universitario Campus Bio Medico, Rome, Italy; ^6^ Hematology Unit, San Bortolo Hospital, Vicenza, Italy, ulssvicenza.it; ^7^ Hematology Unit, Department of Oncology and Hematology, Santo Spirito Hospital, Pescara, Italy; ^8^ Department of Medicine and Aging Sciences, University of Chieti-Pescara, Chieti, Italy, unich.it; ^9^ Haematology Unit, ASL ROMA 1 Santo Spirito Hospital of Rome, Rome, Italy; ^10^ Haematology, Giovanni Paolo II Hospital, Bari, Italy; ^11^ Division of Hematology, Cardarelli Hospital, Naples, Italy, naplesldm.com; ^12^ Hematology and Stem Cell Transplantation Unit, IRCCS National Cancer Institute “Regina Elena”, Rome, Italy; ^13^ Hematology Unit, Fabrizio Spaziani Hospital, Frosinone, Italy; ^14^ Hematology and Transplant Unit, Santa Maria Goretti Hospital, AUSL, Latina, Italy, asl.latina.it; ^15^ GIMEMA Foundation, Rome, Italy

**Keywords:** COVID-19, multiple myeloma, nirmatrelvir/ritonavir, remdesivir, vaccines

## Abstract

The COVID‐19 pandemic posed significant challenges for patients with hematological malignancies, including multiple myeloma (MM), who experienced increased hospitalization and mortality rates due to frailty and immune suppression. This retrospective, multicenter study aimed to evaluate the impact of antiviral therapies and COVID‐19 vaccination doses on clinical outcomes in 206 symptomatic MM patients undergoing active treatment across five Italian regions. The majority (90%) received at least one vaccine dose, and 61% were treated with antiviral agents (nirmatrelvir/ritonavir, molnupiravir, or remdesivir). Of the cohort, 90% were vaccinated, with 89% receiving ≥ 3 doses, and 61% received antiviral agents (nirmatrelvir/ritonavir, molnupiravir, or remdesivir). Patients vaccinated with ≥ 3 doses showed significantly reduced infection severity (*p* = 0.044), hospitalization rates (*p* = 0.008), and infection resolution time (*p* = 0.037). Unvaccinated patients without antiviral therapy had a significantly higher mortality rate (*p* = 0.004). Antiviral therapy, especially when combined with ≥ 3 vaccine doses, further shortened infection resolution time (*p* = 0.011) but did not significantly affect hospitalization rates or disease severity. This study highlights the critical role of ≥ 3 vaccine doses combined with antiviral therapy in improving COVID‐19 outcomes in MM patients, with a reduced infection duration and lower progression to severe disease. Further investigation is required to optimize treatment strategies in this vulnerable population.

## 1. Introduction

In December 2019, a cluster of pneumonia cases was reported in China in the city of Wuhan [1]. This disease rapidly spread across China and many other countries around the world [2]. A new coronavirus was identified as the cause of the disease, and the World Health Organization (WHO) proposed the name of coronavirus disease 2019 (COVID‐19) for the disease and the name of severe acute respiratory syndrome coronavirus 2 (SARS‐CoV‐2) for the many symptoms associated with this new pneumonia [3].

The rapidly expanded COVID‐19 pandemic significantly impacted all areas of daily life. In particular, delivering therapy to patients with hematological malignancies was challenging because serious complications in these patients were reported in up to 63% of cases, with a mortality rate reaching 30%, resulting in a higher risk of death in patients with hematological malignancies diagnosed one year prior to COVID‐19 infection [4–6].

In patients with multiple myeloma (MM), in addition to an increased hospitalization and mortality rate due to frailty, COVID‐19 infection indirectly influenced prognosis because of the discontinuation and/or delay of treatment for a variable period of time [7]. Moreover, as a consequence of the induced immune suppression by disease status and treatment, MM patients had a reduced vaccination response with a lower seroconversion rate. Furthermore, they also had a delay in the viral negativization. [8–10]. Therefore, in order to prevent COVID‐19 infection and reduce the risks associated with its progression as well as its sequelae, in addition to the mandatory vaccination, a series of therapies have been proposed for MM patients [8, 11, 12].

Monoclonal antibodies were introduced early in the pandemic for high‐risk individuals, including MM patients; however, their effectiveness declined markedly with Omicron subvariants, leading to heterogeneous use across regions and eventual withdrawal of several agents.

In Italy, tixagevimab/cilgavimab was authorized for prophylaxis in selected fragile patients; however, its clinical adoption was variable, and its role in actively infected MM patients remained limited.

Following the COVID‐19 pandemic, the antiviral agents developed for intravenous or oral administration have demonstrated efficacy in suppressing viral replication during the early phase of the disease against Omicron subvariants BA.2.12.1, BA.4, BA.5, BQ.1.1, and/or XBB.1.5.

Despite a significant increase in their use in the last phase of the pandemic [13–16], limited experiences have been reported in the medical literature as for the efficacy of antiviral agents against COVID‐19 infection in MM patients during treatment for their hematological malignancy.

The aim of this retrospective study was to evaluate the impact of the antiviral drugs and vaccination doses in a cohort of MM patients affected by COVID‐19 infection who received or did not receive these agents as for infection resolution and hospitalization time as well as for death.

## 2. Patients and Methods

### 2.1. Nature of the Study

This is a retrospective study dealing with COVID‐19 infection outcome in a cohort of 206 symptomatic (CRAB + ve) MM patients, undergoing treatment for their hematologic malignancy, aged > 18 years, observed between January 1, 2020, and August 31, 2023, in 12 hematologic departments of 5 Italian regions (Lazio, Abruzzo, Campania, Apulia, and Veneto). Out of these 206 patients, 153 patients (74, 3%) were observed in 8 hematologic departments of the Latium region and the remaining 53 (25, 7%) in the remaining Italian regions. All patients had COVID‐19 infection demonstrated by molecular or antigenic swab.

Of the 206 patients observed at the time of COVID‐19 infection, 186 (90%) had received at least one anti–COVD‐19 vaccine dose, while 20 (10%) were not vaccinated. The 21 patients who received < 3 vaccination doses were grouped with the unvaccinated patients. Therefore, the total number of unvaccinated patients was 41.

Patients receiving < 3 vaccination doses (*n* = 21) were grouped with unvaccinated individuals (*n* = 20), as recommended by national health authorities and prior MM COVID‐19 studies, due to the limited protective effect of ≤ 2 doses in immunocompromised patients.

Therefore, the total number of “functionally unvaccinated” patients was 41.

### 2.2. Patients

Patients’ clinical characteristics are summarized in Table 1: Overall, median age was 68 years, with a prevalence of male patients (#118; 57%), 158 (77%) had at least a very good partial remission, and 113 (55%) received anti‐CD38 therapy associated with a proteasome inhibitor (#46 = 22%), IMiDS (#41 = 20%), or others therapies (#6 = 3%). The more utilized vaccine (96%) was BNT162b2 Pfizer/BioNTech.

**TABLE 1:** Study population characteristics.

Patients characteristics	Overall (206)	Vaccinated ≥ 3 doses (165)	Unvaccinated^∗^ (41)	*p* value
Median age at COVID‐19 infection, years (median, range)	68 (39, 92)	68 (39, 89)	68 (42; 92)	0.98
Sex, *n* (%)				0.16
Female	88 (43)	66 (40)	22	
Male	118 (57)	99 (60)	19	
Comorbidities, *n* (%)				0.68
No	87 (42%)	69 (42%)	18 (44%)	
Metabolic and cardiovascular	116 (56)	94 (57)	22 (54)	
Second tumor	3 (1.5)	2 (1.2)	1 (2)	
Moderate–severe chronic renal failure	17 (8.3)	13 (8)	4 (10)	0.75
Type of treatment response at onset, *n* (%)				0.36
sCR	104 (51)	85 (52)	19 (46)	
RC	42 (20)	32 (19)	10 (24)	
VGPR	12 (6)	10 (6)	2 (5)	
Stable or progressive disease	21 (10)	14 (8)	7 (17)	
Not yet evaluated	27 (13)	24 (15)	3 (7)	
Type of anticancer treatment, *n* (%)				0.46
Anti CD38 monoclonal antibody	113 (55)	90 (55)	23 (56)	
Proteasome inhibitors	46 (22)	40 (24)	6 (15)	
IMiDs	41 (20)	31 (19)	10 (24)	
Others	6 (3)	4 (2)	2 (5)	
Antiviral therapy, *n* (%)	126 (61)	99 (60)	27 (66)	0.59
Oral	106 (84)	85 (86)	21 (78)	1
Nirmatrelvir/ritonavir	80 (75)	68 (80)	12 (29)	
Molnupiravir	26 (25)	17 (20)	9 (22)	
Intravenous (remdesivir)	20 (16)	16 (14)^∗∗^	6 (22)	0.40
Symptoms of COVID‐19 infection at onset, *n* (%)				< 0.0001
Yes	57 (28)	41 (25)	16 (39)	
No	149 (72)	124 (75)	25 (61)	
Length of viral shedding (days)	14 (4, 152)	13 (4, 152)	15 (4, 48)	0.60
Hospitalization (%)	44 (21)	29 (18)	15 (37)	0.011
Death (%)	13 (7)	7 (4)	6 (14)	0.025

^∗^Among these patients, there are patients who did not receive at least 3 doses of vaccine.

^∗∗^Out of these 16 patients, 2 received also oral antiviral drugs.

As for medical history, 116 patients (56%) had metabolic and/or cardiovascular comorbidities, and 17 patients (8.3%) had renal failure with an estimated glomerular filtration rate (eGFR) less than 30 ml/min × 1.73 m^2^. COVID‐19–directed monoclonal antibodies (casirivimab/imdevimab, sotrovimab, and bebtelovimab) were not included in this analysis. Their use was limited and heterogeneous across centers; moreover, their use was largely restricted to the early pandemic phases before Omicron subvariants rendered them less effective. Because of the extremely small number of MM patients exposed to these agents and the absence of uniform documentation, they were excluded from statistical evaluation. Patients who received pre‐exposure prophylaxis with tixagevimab/cilgavimab (Evusheld) were excluded from the primary analysis because the treatment was not administered for active COVID‐19 infection and could confound evaluation of antiviral efficacy. Only a minimal number of patients across participating centers had received prophylaxis, precluding meaningful subgroup analysis. Patients were stratified according to vaccination status: 186 (90%) patients received a vaccine, while 20 (10%) patients refused vaccination for personal choice. We also stratified patients in terms of the number of vaccination doses: 21/186 patients (11%) received less than 3 doses of vaccine, while 165/186 (89%) received ≥ 3 doses. However, as previously noted, patients who received < 3 vaccination doses were grouped with those who were not vaccinated. Antiviral therapy was received by 126 (61%) patients, of whom only 22 (17%) received intravenous antiviral treatment. The type of antiviral treatment was decided according to local policy, taking into account comorbidities, drug interference, renal function, and pulse oximetry.

Antiviral allocation followed Italian Medicines Agency (AIFA) authorizations and real‐world drug availability, which varied during the study period.

Nirmatrelvir/ritonavir (NIR/r) was used as first‐line therapy when not contraindicated (e.g., major drug–drug interactions). Molnupiravir (MOL) was administered when NIR/r was unsuitable due to comorbidities or medication interference. Due to temporary supply limitations, treatment choice was sometimes constrained by local availability.

NIR/r was consistently considered the first choice of treatment (Table 1). Antiviral treatment was initiated within 5 days of symptom onset or positive swab test. The recommended dosage of NIR/r was 300 mg nirmatrelvir (150 mg twice a day) with 100 mg of ritonavir (100 mg twice a day) orally for 5 days. For MOL, the recommended dosage was 800 mg twice a day for 5 days; it is worth noting that starting from February 2024, the AIFA suspended the marketing of this drug. All patients on oral antiviral treatment followed the instruction concerning oral administration of the drug, potential adverse events, and symptoms monitoring. The majority of patients treated with intravenous remdesivir were hospitalized, and the drug was administered at a dosage of 200 mg (loading dose) on the first day, following by 100 mg once daily on subsequent days for a treatment duration of 5 days.

Remdesivir was administered according to AIFA indications in two regimens:

• a 3‐day outpatient regimen (200 mg on Day 1, then 100 mg on Days 2–3) for patients without radiologic evidence of pneumonia;
• a 5‐day inpatient regimen for patients with confirmed COVID‐19 pneumonia or oxygen requirement.

Due to the retrospective multicenter design, detailed documentation on treatment duration was incomplete for several patients. For this reason, remdesivir‐treated cases were analyzed collectively, and stratification by the 3‐day vs. 5‐day regimen was not feasible.

During antiviral treatment, all other antiviral drugs were discontinued.

### 2.3. Statistical Analysis

Patient characteristics were summarized using frequencies and percentages for categorical variables, while continuous variables were described using medians and quantiles. Univariate analyses were conducted to compare groups, with nonparametric tests employed as appropriate: the chi‐square test or Fisher’s exact test for categorical variables and the Mann–Whitney *U* test or Kruskal–Wallis test for continuous variables.

To evaluate the association between demographic and clinical parameters with outcomes such as infection resolution, clinical worsening, and mortality, both univariate and multivariate regression models were applied. The results of these models were reported as beta coefficients for linear regressions or odds ratios (ORs) for logistic regressions, along with their corresponding 95% confidence intervals (CIs).

All statistical tests were two‐sided, and a *p* value of less than 0.05 was considered statistically significant. Statistical analyses were performed using the R software.

## 3. Results

Symptoms of COVID‐19 were observed only in 57/206 patients (28%). Overall, the median infection resolution time was 14 days (range 4–152). As for hospitalization, 44 (21%) patients were hospitalized, and 13 (7%) died from the infectious disease. Antiviral drugs for treating COVID‐19 infection were administered to 126 patients (61%), while 80 (39%) did not receive antiviral drugs. Out of the 126 patients who received antiviral drugs, 106 (84%) received oral antivirals (NIR/r or MOL) and 20 (16%) received the intravenous drug (remdesivir). Among the latter 20 patients, 2 received oral and intravenous antiviral drugs because of worsening of their clinical conditions.

As for the vaccination status, 186 (90%) patients were vaccinated; of these, 165 (89%) received at least 3 doses and 21 (11%) less than 3 doses and were therefore grouped with the unvaccinated patients. Out of the 165 vaccinated patients, 99 (60%) received antiviral drugs: 85 (86%) oral and 16 (14%) intravenous (Table 1).

Among the 41 unvaccinated patients, 27 (66%) received antiviral therapy, 21 (78%) oral, and 6 (22%) intravenous. Among these patients, 15 (37%) were hospitalized, while 6 (14%) died.

### 3.1. Outcome of COVID‐19 Infection

#### 3.1.1. Role of Vaccinations in the Outcome of These Patients (Figure 1)

As for the influence of the vaccination on the outcome of patients, comparing those who received ≥ 3 doses with those who were unvaccinated, we observed a statistically significant better outcome among those who received ≥ 3 vaccinations.

**FIGURE 1:**
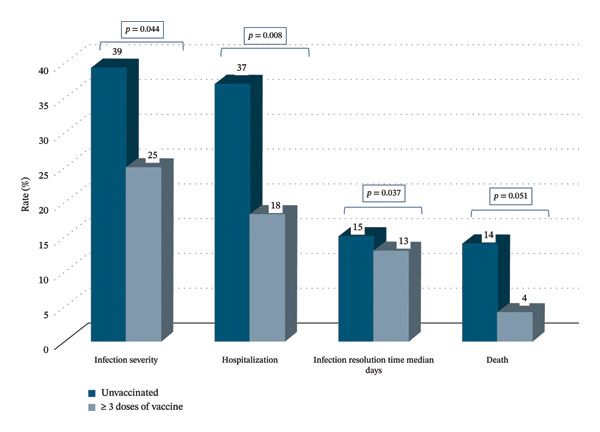
Outcome of COVID‐19 infection by vaccination. Comparison of patients who received ≥ 3 doses of vaccine (grey bars) versus unvaccinated patients (blue bars) in terms of infection severity, hospitalization rates, infection resolution time, and mortality. Patients who received ≥ 3 vaccine doses demonstrated significantly better outcomes compared to unvaccinated patients. Specifically, differences were statistically significant for infection severity (*p* = 0.044), hospitalization rates (*p* = 0.008), and infection resolution time (*p* = 0.037). Mortality rates were lower in vaccinated patients than in unvaccinated patients, though this difference did not reach statistical significance (*p* = 0.051).

In particular, infection severity, hospitalization rates, and infection resolution time were *p* = 0.044, *p* = 0.008, and *p* = 0.037, respectively.

As for the death rate, vaccinated patients had a lower death rate compared to unvaccinated patients, even though not significant (*p* = 0.051).

#### 3.1.2. Outcome of Patients by Antiviral and Vaccination Combination (Table 2)

The unvaccinated patients, who did not receive antiviral therapy, had a statistically significant worse mortality rate (*p* = 0.004), while a statistically significant shorter infection resolution time was observed in those vaccinated patients treated with antiviral drugs (*p* = 0.011).

**TABLE 2:** Outcome of COVID‐19 infection by antiviral drugs and vaccination.

Characteristic	No antiviral			Antiviral		
	Unvaccinated #14	Vaccinated #66	*p* value^1^	Unvaccinated #27	Vaccinated #99	*p* value^1^
Infection severity *n* (%)	6 (42.8%)	16 (24%)	0.087	10 (37%)	25 (25%)	0.22
Hospitalization, *n* (%)	5 (35.7%)	7 (11%)	**0.016**	10 (37%)	22 (22%)	0.13
Infection resolution time, median (range)	15 (5, 21)	15 (5, 40)	0.99	16 (4, 48)	11 (4, 152)	**0.011**
Unknown	3	2		3	2	
Death, *n* (%)	4 (28.5%)	2 (3.0%)	**0.004**	2 (7.4%)	5 (5.1%)	> 0.99

*Note:* Bold values indicate statistically significant results (*p* < 0.05). ^1^Fisher’s exact test; Wilcoxon rank sum test.

## 4. Discussion

In this retrospective multicenter study dealing with the influence of vaccination doses and antiviral treatment on the outcome of COVID‐19 infection in responding MM patients, results highlight the critical role of vaccination doses and antiviral treatment in improving patient outcomes.

In particular, patients who were considered unvaccinated in this study had a higher mortality rate that was statistically significant (*p* = 0.004). Moreover, COVID‐19 infection resolution time was shorter and statistically significant (*p* = 0.011) among patients vaccinated with ≥ 3 doses and who received antiviral drugs. This conclusion is in contrast with that of Pimpinelli et al. [17] who demonstrated limited immunological protection by the vaccine in MM patients, especially in patients treated with anti‐CD38 drugs; moreover, the study of Pimpinelli et al. was also confirmed by Bitoun et al. [18], who conducted a case–control study to compare antispike IgG response and neutralizing activity of anti–SARS‐CoV‐2 antibodies in healthy controls versus MM patients, showing diminished levels of anti‐spike IgG levels compared to controls but with a high proportion of patients achieving a humoral response (89% vs. 97% in controls). Furthermore, Ludwig et al. [8] described in their review several case–control studies that demonstrated a poor response to vaccination in MM patients compared to the general population, particularly in elderly patients, those undergoing advanced lines of therapy, and/or those with uncontrolled disease. The difference between our results and those mentioned above may be attributed to the fact that the majority of patients in our cohort (158/206 = 77%) were at least in very good partial response (VGPR). Moreover, our study was conducted when vaccines and antiviral drugs were available for all patients. This peculiarity is also reflected by a lower mortality rate (13/206 = 7%) observed in our population compared to that reported in the literature [5].

This study also found a weak correlation between new drugs, such as anti‐CD38, IMiDs, and proteasome inhibitors, and the outcome of COVID‐19 infection in MM patients, consistent with the findings previously reported by Krejci et al. [19].

As for the role of antiviral therapy, medical literature reports an important role for it in the outcome of COVID‐19 infection [20]. This result was confirmed by our study where antiviral treatment played an important role in reducing the infection resolution time when it was associated with ≥ 3 vaccination doses. Moreover, those unvaccinated patients who did not receive antiviral treatment had a higher mortality rate (*p* = 0.004). These observations pointed out the important role of antiviral treatment when combined with ≥ 3 vaccination doses.

Therefore, in our study, we observed a significant advantage in patients who received ≥ 3 doses of vaccine compared to those who were considered unvaccinated. Regardless of the type of antiviral drugs administered, Lee et al. [16] in their systematic review and meta‐analysis confirmed enhanced vaccine efficacy in immunocompromised patients who received multiple doses. This finding challenges the prevailing notion that immunocompromised individuals, particularly those with hematologic malignancies, exhibit reduced seroconversion rates following vaccination.

Focusing on patients who received ≥ 3 vaccination doses, our study demonstrated a significantly reduced progression to severe COVID‐19 infection (*p* = 0.044), lower hospitalization rates (*p* = 0.008), and a shorter duration of infection (*p* = 0.037).

Our study also demonstrated a globally low rate of respiratory complications and mortality in patients with MM undergoing active treatment for COVID‐19 infection. These findings are better than those reported in the literature [7].

Finally, the poorer outcomes observed in patients treated with remdesivir may reflect the greater severity of infection in this group, who necessitated hospitalization and intravenous therapy. However, remdesivir prescription is allowed in cases of oxygen‐dependent COVID‐19 pneumonia, thus enclosing patients with more severe infection and a more complex clinical course [21].

In conclusion, from our retrospective study, vaccination with ≥ 3 doses significantly reduced infection severity (*p* = 0.044), hospitalization (*p* = 0.008), and infection resolution time (*p* = 0.037) (Figure 1). Moreover, the use of antiviral drugs combined with ≥ 3 vaccination doses significantly reduced the infection resolution time (*p* = 0.011), even though the use of antiviral drugs did not influence the hospitalization rate and the infection severity.

### 4.1. Study Limitations

This study has several limitations. Its retrospective and multicenter nature inevitably introduces variability in clinical documentation and treatment allocation. The use of COVID‐19–directed monoclonal antibodies was not assessed, as only a minimal number of MM patients received these therapies and their adoption differed considerably among centers, particularly after the emergence of Omicron subvariants that markedly reduced their neutralizing activity. Patients who underwent tixagevimab/cilgavimab prophylaxis were also excluded to prevent potential confounding in the evaluation of infection outcomes. Antiviral allocation may have been affected by AIFA regulations, drug–drug interaction profiles, renal function, and periods of limited availability, potentially reducing comparability across treatment groups. In addition, the duration of remdesivir therapy could not be consistently retrieved, and for this reason, all remdesivir‐treated patients were analyzed collectively. Finally, although multivariate analyses were performed, propensity score matching was not implemented, as it would have substantially reduced the number of evaluable patients and increased the risk of overfitting.

## Author Contributions

Conceptualization and writing–review and editing, Annibali O., M. Di Cecca, and Rigacci L.; data curation, M. Di Cecca, Annibali O., Tomarchio V., Bongarzoni V., Garzia M., Fazio F., Petrucci M. T., Barilà G., Liberatore C., Rago A., Rossini B., Rocco S., Pisani F., De Padua L., Fiori L., Tafuri M. T., Anaclerico B., Cardarelli L., Scomazzon E., Fioritoni F., Caravita Di Torritto T., Quinto A. M., Palmieri S., Lamanda M., and Tamburini A.; formal analysis, Annibali O.; statistical analysis, Piciocchi A.; investigation, methodology, and writing–original draft, Annibali O. and M. Di Cecca.

## Funding

This research received no external funding.

## Disclosure

All authors have read and agreed to the published version of the manuscript.

This study was previously presented in part as a poster at the European Myeloma Network (EMN) Meeting 2023 and published in the abstract book (HemaSphere).

## Ethics Statement

The study was conducted in accordance with the Declaration of Helsinki and approved by the Ethics Committee of Fondazione Policlinico Campus Bio‐medico di Roma (Prot. PAR: 002.22(98.21) OSS). Written informed consent has been obtained from all subjects involved in the study to publish this paper.

## Conflicts of Interest

The authors declare no conflicts of interest.

## Data Availability

The data that support the findings of this study are available from the corresponding author upon reasonable request.
